# The sampling strategy of oral microbiome

**DOI:** 10.1002/imt2.23

**Published:** 2022-05-13

**Authors:** Hongye Lu, Peihui Zou, Yifei Zhang, Qian Zhang, Zhibin Chen, Feng Chen

**Affiliations:** ^1^ Stomatology Hospital, School of Stomatology, Zhejiang University School of Medicine, Zhejiang Provincial Clinical Research Center for Oral Diseases, Key Laboratory of Oral Biomedical Research of Zhejiang Province Cancer Center of Zhejiang University Hangzhou China; ^2^ Department of Periodontology, National Engineering Laboratory for Digital and Material Technology of Stomatology, Beijing Key Laboratory of Digital Stomatology Peking University School and Hospital of Stomatology Beijing China; ^3^ Central Laboratory, Peking University School and Hospital of Stomatology & National Clinical Research Center for Oral Diseases & National Engineering Laboratory for Digital and Material Technology of Stomatology & Beijing Key Laboratory of Digital Stomatology Beijing China

**Keywords:** oral cavity, microbiome, saliva, plaque, sampling strategy

## Abstract

There are multiple habitats in the oral cavity with bacteria, fungi, viruses, and protozoa residing in, which together constitute the oral micro‐ecosystem. These microflorae in the oral cavity primarily include saliva, supragingival dental plaque, subgingival dental plaque, submucosal plaque around implants, plaque in root canals, and plaque on the mucosal surface. The interest and knowledge of the microbiome have dynamically increased with the advancement of technology. Therefore, a reliable, feasible, and practical sampling strategy for the oral microbiome is required for the investigation. This paper introduced the sampling strategy of oral microorganisms, consisting of sample collection, transport, processing, and storage. The materials and devices involved in this study are all commonly used in clinical practice or laboratory. The feasibility and reliability of the sampling methods described in this paper have been verified by multiple studies.

## Highlights

- Oral microbiome attracts substantial attention, and a reliable, feasible, and practical sampling strategy for the oral microbiome is required.
- The habitats of microorganisms in the oral cavity primarily include saliva, supragingival area, subgingival/submucosal area, infected root canals, and mucosal surface.
- The sampling strategy of oral microbiota is described in this study, which consists of sample collection, transport, processing, and storage.

## Graphical abstract

The sampling sites in the oral cavity primarily include saliva, supragingival area, subgingival/submucosal area, infected root canals, and mucosal surface.

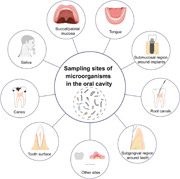

## INTRODUCTION

The oral cavity is home to a wide variety of microorganisms, making it the second‐largest microbial community in the human body, next to the gut [1]. Oral microbiota is comprised of bacteria, fungi, viruses, and protozoa, of which bacteria are the dominant component. The microorganisms differ among individuals and alter dynamically under different clinical conditions, microenvironments, and other influencing factors. It is deeply involved in oral health, even systemic health. Multiple studies have confirmed microorganisms to be the etiological factor of a variety of oral diseases, such as periodontitis [2], caries [2], and mucositis [3]. It is also considered to have a potential association with a number of systemic diseases. Oral microorganisms may play a role in the development of diabetes [4], cardiovascular diseases [5], cancer [6], Alzheimer's disease [7], and other disorders. In turn, oral microorganisms may be influenced by systemic factors to some extent [8].

Despite the fact that oral microbiota has been studied for decades, it still attracts considerable critical attention. Technology has dramatically increased our understanding of the morphological traits, composition, function, and interacting mechanism of oral microorganisms, allowing us to better explore the characteristics of the oral microbial community [9]. High‐throughput sequencing, for example, could provide a global view of the composition of microbial community [10]; fluorescence in situ hybridization (FISH) could present the spatial structure of plaque [11]; proteome and metabolism could help to reveal the metabolites of microorganisms; advanced laboratory techniques are available to investigate their function and pathogenicity. With the advancement of technology, the pathogenic theories of periodontitis, peri‐implantitis, caries, and mucosal disorders have also altered. The developments in oral microbiology have heightened the requirement for the sampling strategy of the oral microbiome.

Every microenvironment accommodates a specific microbial community; therefore, different samples type could provide distinct information. Saliva comes from a variety of sources and immerses practically all the surfaces of hard and soft tissue. It could be used to investigate multiple oral diseases, as well as some systemic diseases. On the surfaces of oral hard and soft tissue, microorganisms exist as biofilm, which forms a sticky deposit on the surface of teeth called dental plaque. Supragingival dental plaque could be used to investigate the microbiota associated with the external tooth surface. Subgingival/submucosal dental plaque could aid in exploring the pathogenesis of periodontal/peri‐implant diseases. Dental plaque in root canals could provide information on canal infection or periapical infection. The samples on the oral mucosa may facilitate the investigation of mucosal diseases.

## THE SAMPLING METHOD FOR SALIVA

Saliva is an affluent body fluid, containing water, minerals, electrolytes, enzymes, cytokines, immunoglobulins, as well as shed cells, food residues, and microbes [12]. Healthy adults produce 600–700 ml of saliva per day [13], and it covers all the surfaces of the oral cavity, including teeth, gingiva, tongue, palate, and mucosa. Saliva acts as a “reservoir” of oral microbiota, collecting microorganisms from multiple habitats and accommodating them. Saliva, in turn, transfers and releases microorganisms into these environments. Saliva is a noninvasive and easily accessible biofluid that permits the investigation of the oral microbiome.

The saliva flow rate fluctuates during the day, and diet and oral hygiene measures have an impact on the salivary microbiome. Saliva flow decreases when sleeping and progressively increases after waking up in the morning [14]. Therefore, collecting samples before cleaning teeth in the morning or between meals is recommended. Salivary flow is classified into two types: unstimulated saliva and stimulated saliva. Unstimulated saliva is obtained under physiological and natural conditions, whereas stimulated saliva is obtained following masticatory stimulation or gustatory stimulation. Stimulated saliva was initially used to evaluate the function of salivary glands. The differences in microbial composition between stimulated and unstimulated saliva have been reported [15, 16]. Considering that additional stimulating substances may have impacts on salivary microorganisms, unstimulated saliva is generally recommended for the investigation of oral microorganisms.

### Materials and devices

Cup container; syringe; stimulant (gum base, cotton puff, rubber bands, sour candy, citric acid, or video), ice box or drikold bucket; sterile centrifuge tube; centrifuge; pipettor; ultra‐low temperature freezer.

### Collection of saliva

There are four methods for collecting unstimulated saliva:

*Method 1*: Ask the volunteer to hold the sterile containers; sit still, head down, mouth slightly open, eyes open, head slightly forward. Avoid swallowing and allow saliva to flow naturally into the container [17].

*Method 2*: Ask the volunteer to sit still with his/her head up, and avoid swallowing. Concentrate the saliva on the floor of the mouth before spitting them into a container [17].

*Method 3*: Ask patients to sit on the dental chair and open their mouths. Suction off the saliva that accumulates in the mouth with a syringe.

*Method 4*: The whole saliva can also be sampled by placing sterile absorbent cotton or other absorbent materials in the mouth. Then, squeeze the saliva into the container.

Collecting unstimulated 1‐ml saliva typically takes 5–10 min. Collecting saliva with absorbent material should avoid wiping away the plaque on the mucosal surfaces.

There are three methods for collecting stimulated saliva:

*Method 1*: Ask volunteers to chew stimulants, such as paraffin wax [15, 18], unflavored chewing gum base, cotton puff, or rubber bands for 30 s. Then ask them to spit the accumulated saliva into the container (mastication stimulation) [17].

*Method 2*: Drop the sour candy or 4% citric acid on the tongue dorsum and wait for 1 min. Ask the volunteer to spit the accumulated saliva into the container (gustatory stimulation) [19].

*Method 3*: Watching a video of acidic foods or imagining some acidic foods can also help while collecting saliva. Ask the volunteer to spit the accumulated saliva into the container (imaginary stimulation).

Collecting stimulated 1‐milliliter saliva typically takes 1–5 min.

### Sample transport and processing

The samples could be put into an ice box or drikold bucket to transfer, preferably within 3 h. The collected saliva was separated into a 1.5 ml sterile centrifuge tube and centrifuged (10,000–16,000 g, 4℃, 15 min). The supernatant was removed and sediment was kept for further detection [20].

### Sample storage

**Figure 1:**
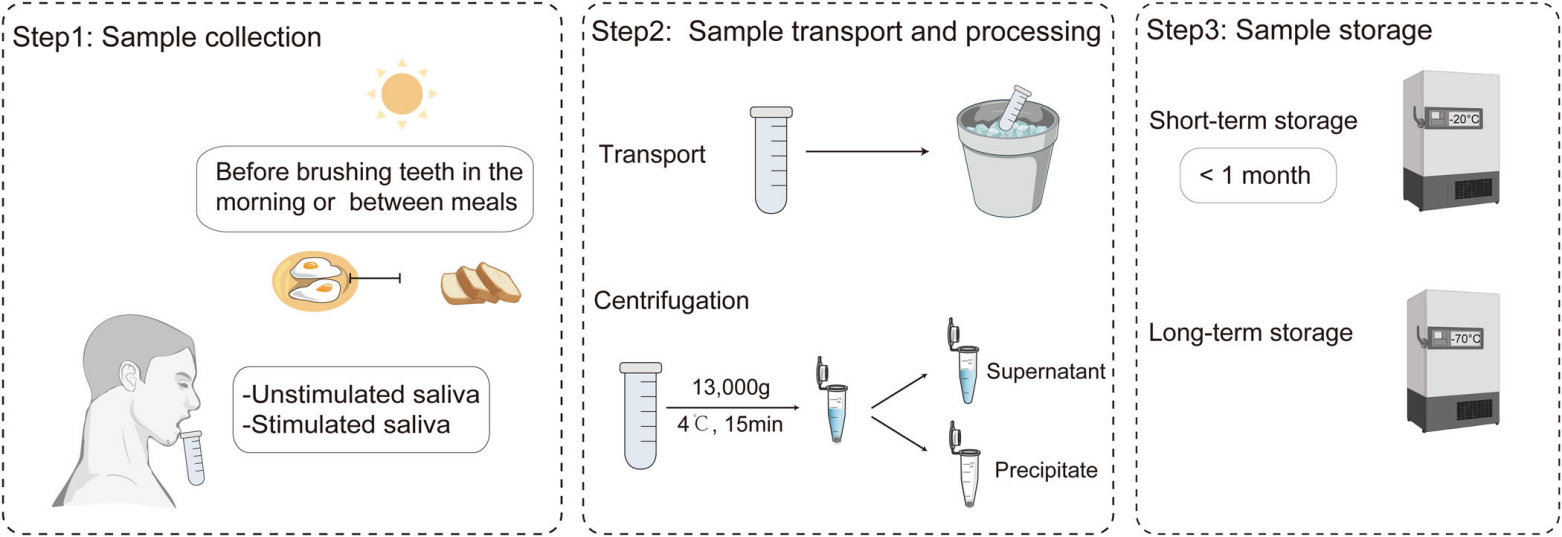
Sampling process of saliva.

The samples should be sent for subsequent detection and analysis as soon as possible. If an immediate analysis is not available, the samples could be stored at −80°C ultra‐low temperature freezer or temporally stored at −20°C for 1 month. To harvest DNA from bacteria, centrifuge and form a bacterial pellet before freezing [20]. A cryoprotective agent (such as 80% glycerol [21] or 10%–20% skim milk [22]) should be added if the sample is kept for subsequent bacteria culture (Figure 1).

## THE SAMPLING METHOD OF SUPRAGINGIVAL DENTAL PLAQUE

The bacteria colonize on the tooth surface with the medium acquired pellicle, which is predominantly generated by salivary proteins and glycoproteins [23]. The supragingival dental plaque is an ecosystem existing as a biofilm on the tooth, which is influenced by various factors, consisting of food, mastication, muscular activity, saliva rinsing, and oral hygiene measures [24]. The buildup of biofilm and the accumulation of acidogenic microorganisms are widely acknowledged as the etiology of caries [25]. Dental plaque produces acids from carbohydrates, which leads to caries. *Mutans Streptococci* have been identified as the primary pathogens of dental caries, and they are thought to induce caries by generating acid and water‐insoluble glucan [26]. The proportions and numbers of acid‐base‐producing bacteria are considered the core of dental caries activity [27]. The analysis of supragingival microorganisms could facilitate the investigation of oral health.

### Materials and devices

Oral cavity check‐up trays with forceps, probe, and mouth mirror; sterile cotton rolls or rubber dam; sample collector on the tooth surface (sterile probe, swab, or scraper); sample collector in the tooth cavity (sterile probe, curette, excavator, floss, or fine wire); sterile centrifuge tube; buffer solution (RNA protect reagent, TE buffer, Tris–HCl, EDTA, normal saline, PBS, or double‐distilled water); ice box or drikold bucket; centrifuge; pipettor; ultra‐low temperature freezer.

### Sample collection

The sampling procedure is carried out on the dental chair with sterile equipment. Before collecting the sample, rinse the mouth with purified water to removeany food residue left in the mouth.

*Dental plaque on the tooth surface*: ask the volunteer to open his/her mouth, and isolate the sampling site from saliva with sterile cotton rolls or a rubber dam before sampling. Scrape the plaque samples from the target tooth surface using a sterile probe, swab, or scraper. After that, transfer the samples to a 0.6 ml sterile centrifugal tube. Dental plaque can also be collected using a sterile small brush or swab, and then eluted the plaque by scrubbing them in the prepared buffer for about 1 min.

*Dental plaque in caries*: The tool for collecting dental plaque in caries depends on the location and profile of the tooth cavity: sterile probe and curette are generally used to collect dental plaque on the occlusal surface and axial surface; sterile probe, floss, or fine wire can be used on proximal surfaces; sterile curettes can be used for collecting the samples in root caries; sterile excavators could be used in soft and moist cavity. After sampling, elute plaque in prepared buffer (scrub for about 1 min).

The buffers for plaque collection could be RNA protect reagent, TE buffer, 10 mM Tris–HCl, 1 mM EDTA, phosphate‐buffered saline (PBS), normal saline, and double‐distilled water, depending on the research goal. All of these solutions support subsequent preservation and detection of the microbiome. RNA protect reagent, TE buffer, Tris–HCl, and EDTA could protect RNA and DNA from cleavage. Dealt with PBS buffer (pH = 7.4) could facilitate subsequent analysis of protein, cytokines in the supernatant; Dealt with double distilled water allow the subsequent detection of metabolites of microorganisms. In addition, pooled samples of multiple index teeth can be used to investigate patient‐level microbiome. Pooled samples of multiple sites with the same clinical condition can be used to investigate the characteristics of the microbiome correspondingly [28].

### Sample transport and processing

**Figure 2:**
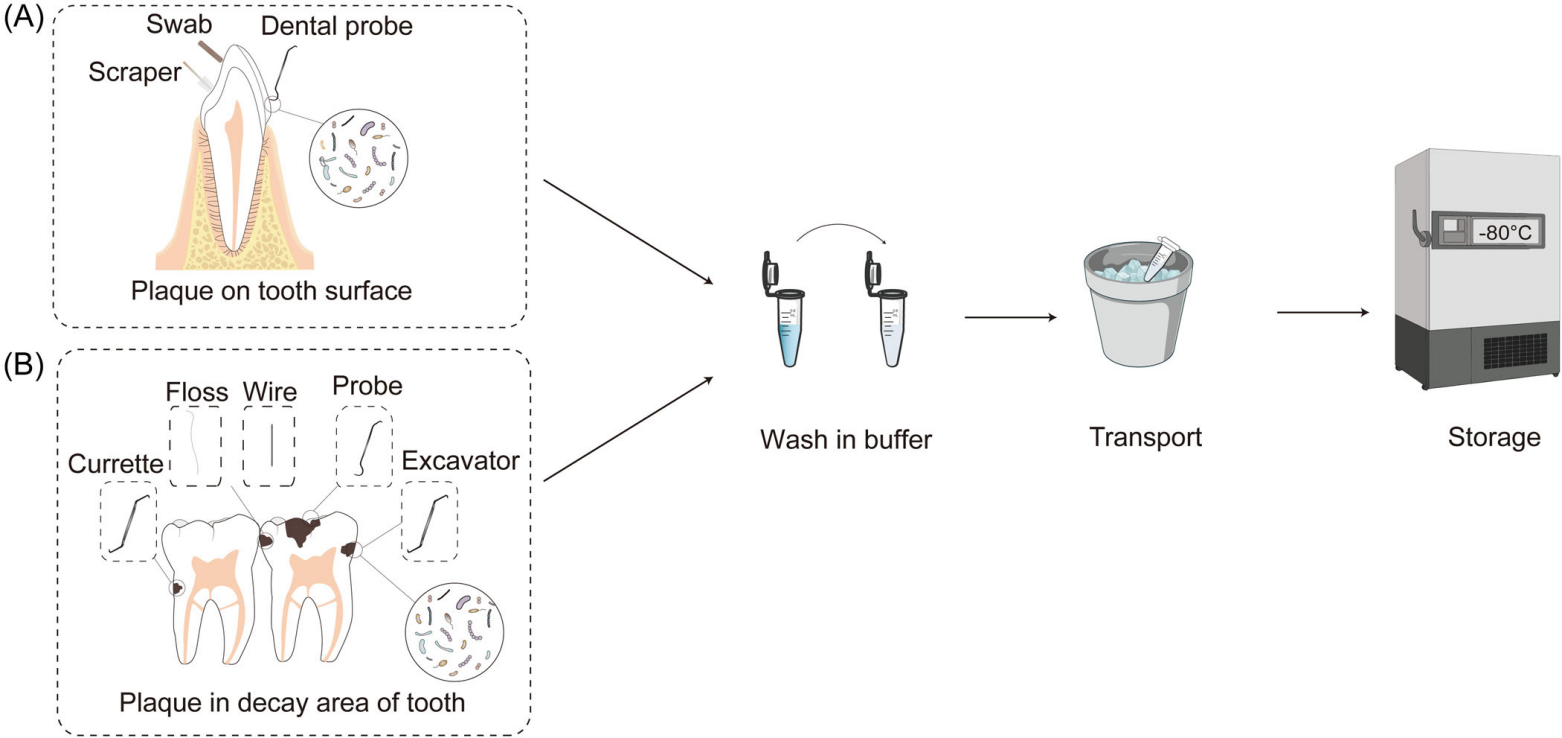
Sampling process of supragingival dental plaque. (A) Supragingival dental plaque on the tooth surface. (B) Dental plaque in the decayed area of the tooth.

The collected samples could be transported to the laboratory within an ice box or drikold bucket. The collected sample should be centrifuged (10,000–16,000 g, 4℃, 15 min) to obtain the sediment for further detection (Figure 2).

### Sample storage

The samples should be sent for subsequent detection and analysis as soon as possible. If an immediate analysis is not available, the samples could be stored at −80°C ultra‐low temperature freezer or temporally stored at −20°C within 1 month. To harvest DNA from bacteria, centrifuge and form a bacterial pellet before freezing [20]. A cryoprotective agent (such as 80% glycerol [21] or 10%–20% skim milk [22]) should be added if the sample is kept for subsequent bacteria culture.

## THE SAMPLING METHOD OF SUBGINGIVAL PLAQUE AROUND TEETH AND SUBMUCOSAL PLAQUE AROUND IMPLANTS

Subgingival dental plaque has been widely accepted as the initial factor of periodontitis for long [29]. The accumulation of plaque also has been identified as the etiological factor of peri‐implant diseases [30]. Based on similar anatomical structure and immunological characteristics, the pathogenic mechanism of periodontal and peri‐implant diseases is comparable [31]. Meanwhile, the inflammatory internal wall of periodontal/peri‐implant pockets opens a window for systemic infection. Therefore, the investigation of the subgingival microbiome and submucosal microbiome could help to evaluate the key pathogenic factor of periodontal diseases and peri‐implant diseases. It could also expedite our understanding of the relationship between oral infection and systemic conditions.

### Materials and devices

Oral cavity check‐up trays with forceps, probe, and mouth mirror; sterile cotton rolls; sterile curette; paper strips/points; 0.6 and 1.5 ml sterile centrifuge tube; buffer solution (RNA protect reagent, TE buffer, Tris‐HCl, EDTA, normal saline, PBS, or double distilled water); ice box or drikold bucket; alcohol lamp; oscillator; centrifuge; pipettor; ultra‐low temperature freezer.

### Sample collection

According to the research design, the sample could be collected at the site level, tooth/implant level, and patient level. Site‐specific samples could provide information on local lesions. Tooth‐/implant‐level investigation generally needs to sample 1–6 sites. The sampling sites are often selected from the mesial and distal buccal sides of the target teeth, which can avoid saliva contamination as much as possible. For a tooth with shallow gingival sulcus or implant with shallow mucosal sulcus, collecting samples from multiple sites could help to obtain a sufficient quantity of microorganisms. A patient‐level investigation needs to pool the samples of several teeth or implants. Ramfjord index teeth are considered to be representative to assess overall periodontal condition [32]. Ramfjord index teeth consist of 16, 21, 24, 36, 41, 44 [32]. If the index teeth are lost, adjacent teeth in the same region will be sampled.

Before sample collection, ask volunteers to rinse their mouths to remove food residue in the mouth. After scraping the calculus, food residue, plaque, and soft debris on the supragingival tooth surface with a probe or scaler or small cotton ball, the saliva was isolated with sterile cotton around sampled sites.

**Figure 3:**
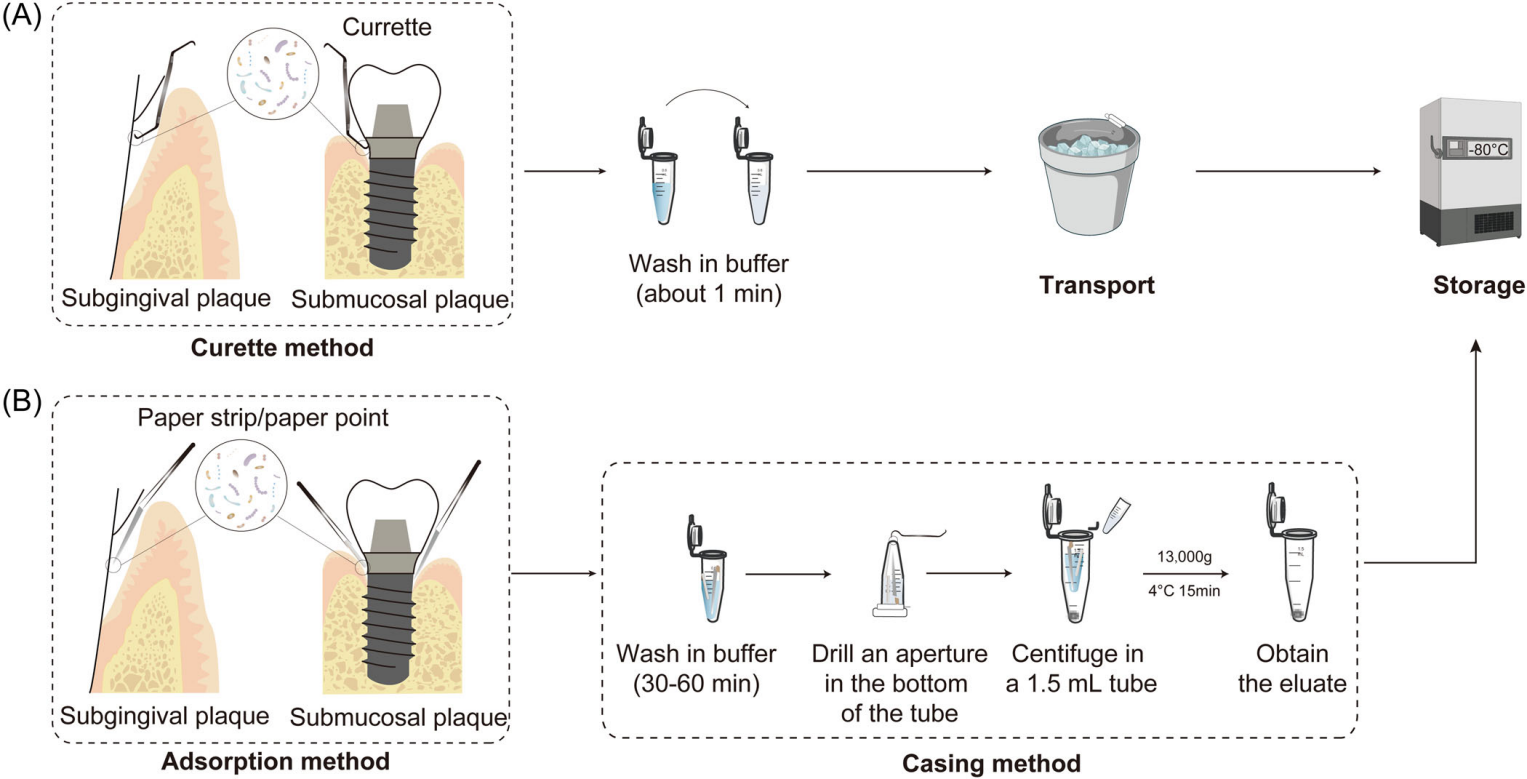
The sampling process of subgingival plaque and submucosal plaque. (A) The sampling of subgingival plaque around the tooth or submucosal plaque around the implant with the curette method. (B) The sampling method of subgingival plaque around the tooth or submucosal plaque around the implant with the adsorption method.

Sampling collection of subgingival plaque or submucosal plaque is carried out on the dental treatment chair with sterile sampling equipment. The commonly used methods for subgingival plaque and submucosal plaque around implants include the curetting method (Figure 3a) and the adsorption method (paper strips, Figure 3b). Both of them are reliable and widely used. However, the curetting method could mainly collect attached plaque on the surface of teeth or implants, while the adsorption method has more advantages in collecting unattached bacteria in periodontal or peri‐implant pockets.

Curette method (plaque method): Subgingival plaque in the periodontal pocket or submucosal plaque around the implant was collected with a sterile periodontal curette and placed in a 0.6 ml sterile centrifugal tube for transport in an ice box or drikold bucket [28, 33].

Adsorption method (gingival crevicular fluid (GCF) method): For subgingival/submucosal sampling, 2 × 10 mm filter paper strips (Whatman, United Kingdom) and 0.02 35# absorbent paper points (GAPADENT, China) with 0.5–1 cm tip cut off is recommended. The strips should be processed using a sterile tweezer to the proper size, and accept high‐pressure steam sterilization and drying before sampling. Hold absorbent paper points or filter paper strips, insert them into the periodontal pockets along the tooth surface, hold on for 30 s when resistance was encountered, and then put it into a 0.6 ml sterile centrifugal tube for transport in an ice box or drikold bucket. Paper strips or paper points should be placed into the bottom of the sulcus or pockets to obtain the plaque at the frontier of disease progress. For healthy peri‐implant tissue with a tight cuff, the curette could be very difficult to insert in without damaging the mucosa. In that case, the adsorption method with finer strips would be a better choice. The paper strips should be carefully inserted into the sulcus or pockets along the margin of the gingiva or mucosa [34, 36].

### Sample processing

The collected samples could be transported to the laboratory within an ice box or drikold bucket.

*Curette method*: The collected sample should be centrifuged (10,000–16,000 g, 4℃, 15 min) to obtain the sediment for further detection.

*Adsorption method*: The samples collected by paper strips or paper points need to be processed by the casing (pipe‐in‐pipe) method. The detailed procedure is as follows: Add 100 μL buffer solution into the 0.6 ml centrifuge tube with sterile filter paper strips or absorbent paper points sample, and vibrate at 4℃ for 30–60 min. Invert the 0.6 ml centrifuge tube, quickly pierce and pull it out with a red‐hot needle tip in the center of the bottom to make a small aperture (<the diameter of filter paper strips or absorbent paper points). Then, put the tube into a 1.5 ml sterile tube and centrifuged symmetrically (10,000–16,000 g, 4℃, 15 min). The supernatant should be gently absorbed and placed into another centrifugal tube for retention, and the precipitation could be obtained as the plaque sample. The sample could also be cryopreserved before sample processing (Figure 3).

### Sample storage

The samples should be sent for subsequent detection and analysis as soon as possible. If an immediate analysis is not available, the samples could be stored at −80°C ultra‐low temperature freezer or temporally stored at −20°C for 1 month. To harvest DNA from bacteria, centrifuge and form a bacterial pellet before freezing [20]. A cryoprotective agent (such as 80% glycerol [21] or 10%–20% skim milk [22]) should be added if the sample is kept for subsequent bacteria culture.

## THE SAMPLING METHOD OF DENTAL PLAQUE IN ROOT CANALS

The colonization of bacteria in the root canal could result in pulpitis [37], periapical periodontitis [38], and even osteomyelitis. The pathogenesis of microorganisms in the root canal system determines the degree of inflammation, symptoms, and clinical treatment effect. Therefore, eliminating bacteria from the infected root canal and preventing reinfection of the tooth is the key point of root canal treatment to preserve the teeth. For prolonged and refractory infections or cases of risk of systemic spread of infection, root canal sampling for microbiological diagnostics is recommended.

### Materials and devices

Oral cavity check‐up trays with forceps, probe, and mouth mirror; sterile cotton rolls or rubber dam; sterile curette; paper points; 0.6 and 1.5 ml sterile centrifuge tube; buffer solution (RNA protect reagent, TE buffer, Tris–HCl, EDTA, normal saline, PBS, or double distilled water); ice box or drikold bucket; alcohol lamp; centrifuge; pipettor; ultra‐low temperature freezer.

### Sample collection

The adsorption sampling method with sterile paper points is usually used for plaque samples in the infected root canal. If there is much exudation, springe with a fine point could also be used to collect the sample. The following operations are carried out on the dental treatment chair with sterile equipment. Before sampling, remove food residue, calculus, plaque, and soft debris from the crown of teeth to avoid potential contamination; cut the canal wall with a root canal preparation instrument to release biofilm. In addition, sample collection requires a clear approach to the medullary cavity and root canal (avoid plaque damage caused by excessive flushing).

Use sterile cotton to isolate sampling sites. Insert sterile hygroscopic paper points (paper points should be chosen according to the taper and thickness of the root canal) into the root canal and retain them for 30 s. Then remove and put them into a 0.6 ml sterile centrifugal tube for transport in an ice box or drikold bucket. The wet cusp of paper strips may indicate that the collection of samples is successful. If the root canal is desiccative making the dental plaque difficult to be absorbed with paper points, we can inject some sterile solution (like saline or double‐distilled water), then extract the fluid in the root canal using syringe or paper points.

### Sample processing

**Figure 4:**
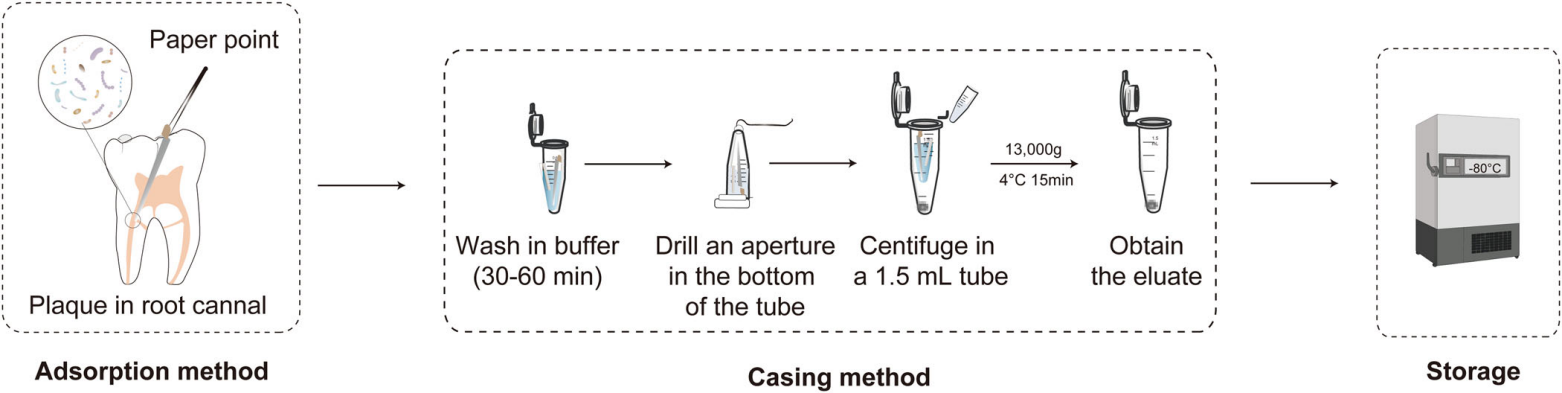
Sampling process of plaque in the root canal.

The casing (pipe‐in‐pipe) method is described above. Add 100 μl buffer solution into a 0.6 ml centrifuge tube with paper strips or paper points, and vibrate for 30–60 min at 4℃. Invert the 0.6 ml centrifuge tube, quickly pierce and pull it out with a red‐hot needle tip in the center of the bottom to make a small aperture (<the diameter of absorbent paper points or filter paper strips). Then, place the above 0.6 ml centrifuge tube in a 1.5 ml sterile tube and centrifuge symmetrically (10,000–16,000 g, 4℃, 15 min). The supernatant should be gently removed and placed into another centrifugal tube for retention, and the precipitation should be obtained as plaque (Figure 4).

### Sample storage

The samples should be sent for subsequent detection and analysis as soon as possible. If an immediate analysis is not available, the samples could be stored at −80°C ultra‐low temperature freezer or temporally stored at −20°C for 1 month. To harvest DNA from bacteria, centrifuge and form a bacterial pellet before freezing [20]. A cryoprotective agent (such as 80% glycerol [21] or 10%–20% skim milk [22]) should be added if the sample is kept for subsequent bacteria culture.

## THE SAMPLING METHOD OF PLAQUE ON THE SURFACE OF THE ORAL MUCOSA

Oral mucosa is laminated squamous epithelium lining the inside of the mouth, consisting of lining mucosa, masticatory mucosa, and special mucosa [39]. Healthy epithelia with cell–cell junction only have straticulate biofilm on them due to the muscle movement and saliva scour, while ulcerated mucosa generally has many microorganisms accumulating on it and infiltrating into tissue [39]. The tongue dorsum harbors abundant nipples on the surface of the epithelium, which provides a favorable microenvironment for microorganisms to colonize and reproduce [40]. In addition to the tongue dorsum, coating mucosa in the oral cavity serves as soft tissue surface, lateral and abdominal sides of the tongue, buccal mucosa, hard palate, soft palate, gingiva, and tonsils. The microorganism on the mucosa is closely related to mucosal health and diseases. Halitosis [41], herpes gingivostomatitis [42], hand‐foot‐and‐mouth disease [43], and oral candidiasis [44] are caused by pathogenic microorganisms, consisting of bacteria, viruses, and fungus. The analysis of the microbiome on the mucosa can also assist in the diagnosis and evaluation of some systemic infections with oral manifestations, such as oral tuberculosis [45] and syphilis [46].

### Materials and devices

Oral cavity check‐up trays with forceps, probe, and mouth mirror; sterile cotton swab, brush, or batten; 0.6 and 1.5 ml sterile centrifuge tube; buffer solution (RNA protect reagent, TE buffer, Tris‐HCl, EDTA, normal saline, PBS, or double‐distilled water); ice box or drikold bucket; centrifuge; pipettor; ultra‐low temperature freezer.

### Sample collection and transport

Sampling sites on oral mucosa mainly include tongue dorsum, buccal mucosa, and palate. Volunteers should rinse their mouths with purified water to remove any food residue before sampling.

*Tongue dorsum*: Ask volunteers to open their mouth and extend their tongue slightly, brush with a sterile brush from one side to the other side in an imbricate shape on the back of the tongue, cut off the head of the brush with a plaque on it, and put it into a sterile tube. A sterile scraper and cotton swab could also be used to collect the plaque on the tongue dorsum [47].

*Other oral coating mucosae (lining mucosa and masticatory mucosa)*: Scrape the plaque on the mucosal surface with a sterile batten or cotton swab [28], put it into the sterile tube, and wash it repeatedly in the tube with buffer solution. The sample should be transported within an ice box or drikold bucket.

### Sample processing

**Figure 5:**
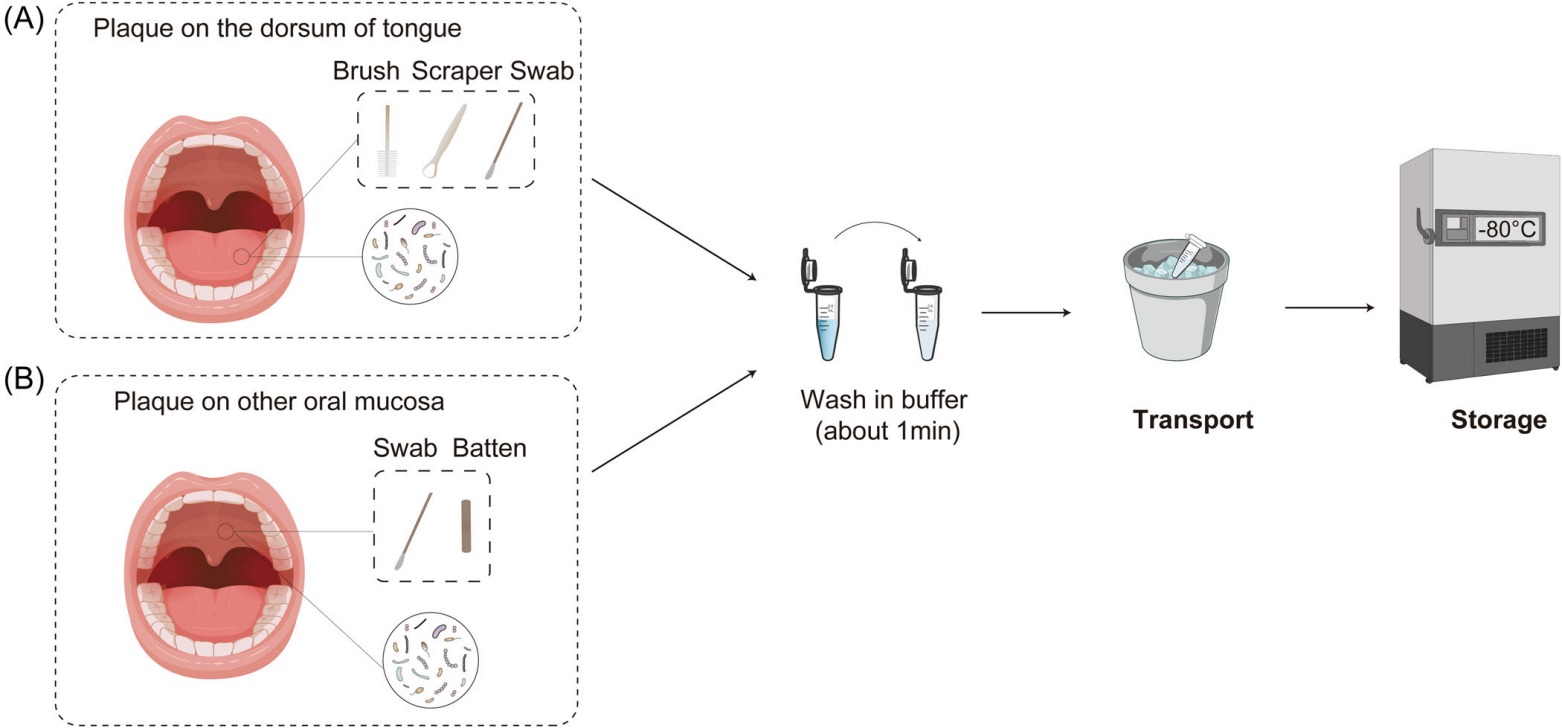
The sampling process of plaque on the oral mucosa. (A) Sampling of plaque on tongue dorsum with a sterile brush, scrape, or cotton swab. (B) The sampling method from other mucosal surfaces with sterile batten or cotton swab.

Add freshly prepared buffer solution. The brush was removed from the liquid, centrifuged at low temperature (10,000–16,000 g, 4℃, 15 min), and the supernatant was gently absorbed and discarded to obtain the precipitate as a tongue dorsal plaque sample (Figure 5).

### Sample storage

The samples should be sent for subsequent detection and analysis as soon as possible. If an immediate analysis is not available, the samples could be stored at −80°C ultra‐low temperature freezer or temporally stored at −20°C within 1 month. To harvest DNA from bacteria, centrifuge and form a bacterial pellet before freezing [20]. A cryoprotective agent (such as 80% glycerol [21] or 10%–20% skim milk [22]) should be added if the sample is kept for subsequent bacteria culture.

## THE SAMPLING METHOD OF OTHER SITES

There are retention, hydrops, effusion, and abscess in the oral cavity, which may be a result of infection. The sample in these sites could be obtained with a sterile syringe or sterile paper strips depending on the quantity and position. Sometimes surgery may be required to create an approach for sample collection.

## SUMMARY

Sampling procedures could be conducted by dentists, dental hygienists, or trained researchers. The most important thing for sample collection is to avoid injuring volunteers. Gingiva is extremely vulnerable and could get hurt during the collection of subgingival/submucosal dental plaque. Another important thing is to avoid potential contamination of the nearby microbial ecosystem. Collecting subgingival/submucosal dental plaque is very easy to be polluted by supragingival dental plaque because of the confined access. Saliva is also very easy to be polluted by plaque from the mucosal surface.

With some microorganisms firmly colonizing the oral cavity serving as the fingerprint, the oral microbiome could reflect individual features to some extent. On the other hand, the oral cavity is a complex and incessantly changing environment, which is also affected by a variety of external factors and systemic factors. Therefore, only the sample in real‐time could provide precise information about the dynamical oral microbiome. The characteristic of the microbiome is influenced by several factors, such as age, gender, circadian rhythm, diet, smoking, physiological status, oral hygiene measures, and intake of medicine. Therefore, investigators should keep an eye on the influencing factors and choose appropriate sampling procedures according to the purpose of the research.

## AUTHOR CONTRIBUTIONS

**Hongye Lu** wrote the manuscript and revised the figures. **Peihui Zou** designed and constructed the diagrams. **Yifei Zhang** and **Qian Zhang** revised the manuscript. **Zhibin Chen** and **Feng Chen** supervised this project. All authors have read the final manuscript and approved it for publication.

## CONFLICTS OF INTEREST

This study was authorized by Bio‐protocol.

## Data Availability

Supplementary materials (figures, tables, scripts, graphical abstract, slides, videos, Chinese translated version, and updated materials) may be found in the online DOI or iMeta Science http://www.imeta.science/.
